# Extended-spectrum *β*-lactamase-producing *E. coli* from retail meat and workers: genetic diversity, virulotyping, pathotyping and the antimicrobial effect of silver nanoparticles

**DOI:** 10.1186/s12866-023-02948-0

**Published:** 2023-08-07

**Authors:** Heba A. Ahmed, Ibrahim Elsohaby, Amina M. Elamin, Abeer E. Abd El-Ghafar, Gamilat A. Elsaid, Mervat Elbarbary, Rasha A. Mohsen, Tamer M. El Feky, Rasha M. El Bayomi

**Affiliations:** 1 Department of Zoonoses, Faculty of Veterinary Medicine, Zagazig University, Zagazig City, 44511 Sharkia Governorate Egypt; 2 Department of Infectious Diseases and Public Health, Jockey Club of Veterinary Medicine and Life Sciences, City University of Hong Kong, Hong Kong SAR, China; 3 Centre for Applied One Health Research and Policy Advice (OHRP), City University of Hong Kong, Hong Kong SAR, China; 4 Department of Animal Medicine, Faculty of Veterinary Medicine, Zagazig University, Zagazig City, 44511 Sharkia Governorate Egypt; 5 Department of Food Hygiene, Zagazig Branch, Agriculture Research Center (ARC), Animal Health Research Institute (AHRI), Zagazig City, Egypt; 6 Department of Bacteriology, Mansoura Branch, Agriculture Research Center (ARC), Animal Health Research Institute (AHRI), Mansoura City, Egypt; 7 Department of Food Hygiene, Mansoura Branch, Agriculture Research Center (ARC), Animal Health Research Institute (AHRI), Mansoura City, Egypt; 8 Department of Food Control, Faculty of Veterinary Medicine, Zagazig University, Zagazig City, 44511 Sharkia Governorate Egypt

**Keywords:** Retail meat, ESBL, *E. coli*, Antimicrobial resistance, Multidrug resistant, Silver nanoparticles

## Abstract

**Background:**

The spread of extended-spectrum *β*-lactamases (ESBL) producing *E. coli* from food animals and the environment to humans has become a significant public health concern. The objectives of this study were to determine the occurrence, pathotypes, virulotypes, genotypes, and antimicrobial resistance patterns of ESBL-producing *E. coli* in retail meat samples and workers in retail meat shops in Egypt and to evaluate the bactericidal efficacy of silver nanoparticles (AgNPs-H_2_O_2_) against multidrug resistant (MDR) ESBL-producing *E. coli*.

**Results:**

A total of 250 retail meat samples and 100 human worker samples (hand swabs and stool) were examined for the presence of ESBL- producing *E. coli*. Duck meat and workers’ hand swabs were the highest proportion of ESBL- producing *E. coli* isolates (81.1%), followed by camel meat (61.5%). Pathotyping revealed that the isolates belonged to groups A and B1. Virulotyping showed that the most prevalent virulence gene was Shiga toxin 2 (*stx2*) associated gene (36.9%), while none of the isolates harbored *stx1* gene. Genotyping of the identified isolates from human and meat sources by REP-PCR showed 100% similarity within the same cluster between human and meat isolates. All isolates were classified as MDR with an average multiple antibiotic resistance (MAR) index of 0.7. AgNPs-H_2_O_2_ at concentrations of 0.625, 1.25, 2.5 and 5 μg/mL showed complete bacterial growth inhibition.

**Conclusions:**

Virulent MDR ESBL-producing *E. coli* were identified in retail meat products in Egypt, posing significant public health threats. Regular monitoring of ESBL-producing *E. coli* frequency and antimicrobial resistance profile in retail meat products is crucial to enhance their safety. AgNPs-H_2_O_2_ is a promising alternative for treating MDR ESBL-producing *E. coli* infections and reducing antimicrobial resistance risks.

## Background

For several decades, *β*-lactam antibiotics have been considered the antimicrobial agents of choice in humans and veterinary medicine. However, the effectiveness of these drugs has been diminished due to the emergence of *β*-lactamase-producing bacteria, particularly within the *Enterobacterales* [1]. Recently, as a result of the increased production of extended-spectrum *β*-lactamases (ESBL) a rise in *β*-lactam resistance in *E. coli* has increased [2]. ESBL-producing *E. coli* or their resistance genes can potentially be transmitted through direct contact, the food chain, or environmental sources [3]. The emergence of ESBL-producing bacteria linked to cattle, poultry, and pigs may be linked to the gradual increase in the usage of third-generation cephalosporins in food animal production [4, 6].

The genome of *E. coli* consists of a mobile gene core that determines the strain pathotypes. Various virulence factors determinants have been attributed to *E. coli* pathogenicity, including Shiga toxin-associated genes (*stx*1 and *stx*2), toxin production genes such as hemolysin (*hly*) and the *ast*A gene encoding enteroaggregative *E. coli* heat-stable enterotoxin, intimin encoding gene (*eae*), and fimbrial H gene (*fim*H). *E. coli* are classified into eight phylotypes (A, B1, B2, C, D, E, F, clade 1) based on the presence or absence of *ChuA*, *yjaA*, *TspE4.C2*, and *arpA* genes [5, 6]. Phylotypes B2 and D are frequently found in humans, with phylotype B2 being associated with extraintestinal disease in both animals and humans [7]. Different properties of isolates are indicated by their typing, which is helpful for identifying the circulation pattern across various sources and hosts [8]. The presence of similarities between clinical and foodborne ESBL isolates suggests that food products can serve as a reservoir for ESBL-producing bacteria and their genes [9, 10, 13].

ESBL enzymes hydrolyze broad-spectrum cephalosporins, including ceftazidime, cefotaxime, cefuroxime, ceftriaxone and cefepime. Most ESBLs are classified as Ambler class A enzymes, which includes the sulfhydryl variable (SHV), Temoneria (TEM) and cefotaxime (CTX-M) types [11]. Unlike other bacterial families, *Enterobacterales* encode SHV, TEM and CTX-M genes on plasmids rather than the chromosome [12], ESBL-producing *E. coli* is becoming more resistant to fluoroquinolones and aminoglycosides, leading to the evaluation of colistin as an important alternative antimicrobial for human therapy [13, 17].However, colistin has been extensively used in veterinary medicine for years to prevent and treat gastrointestinal infections caused by *Enterobacterales* in food producing animals [14]. The *mcr-1* gene, responsible for mobile colistin resistance, has been detected in *Enterobacterales* isolates from humans, food, companion animals, meat, and the environment in various studies [15].

Cephalosporins are widely used to treat various infections caused by both Gram-negative and Gram-positive bacteria [16]. They possess a broader activity range and are less susceptible to inactivation by *β*-lactamase enzymes compared to other *β*-lactam antibiotics, with successive generations of cephalosporins having a wider spectrum of activity [17]. The World Health Organization has prioritized third-generation cephalosporins (such as cefotaxime and ceftriaxone) in monitoring and stewardship programs for antibiotic resistance due to their significance to human health [18, 23].ESBLs, which hydrolyze third-generation cephalosporins, can transmit acquired resistance to other bacterial populations, leading to the spread of antibiotic resistance [12, 19].

In the past, the most common ESBL genes found in *K. pneumoniae* were the allelic variants of TEM-1, TEM-2, and SHV-1 that are the traditional TEM- and SHV-types, exhibit enhanced activity against extended-spectrum cephalosporines, and are primarily found in the hospital environment. However, in recent times, ESBL genes have been more frequently identified in *E. coli*, with the majority belonging to the CTX-M- group [20, 21].

The increasing prevalence of infections caused by MDR bacteria, coupled with the rising problem of acquired resistance resulting from improper antibiotic use, highlights the urgent need to reduce antibiotic consumption by exploring alternative approaches [22]. Among the potential alternatives, nanoparticles have emerged as a promising alternative to antibiotics for controlling infectious agents. Silver nanoparticles (AgNPs) exhibit biocidal effects against various foodborne bacteria [23]. They can interact with the cell surface of Gram-negative bacteria, causing damage and structural changes that enhance bacterial permeability [24]. Therefore, this study aims to (i) determine the occurrence, pathotypes, virulotypes, genotypes, and antimicrobial resistance patterns of ESBL-producing *E. coli* in retail meat samples and workers in contact in Egypt, and (**ii**) evaluate the bactericidal efficacy of silver nanoparticles (AgNPs-H_2_O_2_) against MDR ESBL-producing *E. coli*.

## Materials and methods

### Sample collection

The study received approval from the Zagazig University Institutional Animal Care and Use Committee (ZU-IACUC) under approval number ZU-IACUC/2/F/214/2022. Animal procedures were conducted following the ARRIVE guidelines. A total of 250 meat samples (chicken breast, duck breast, turkey breast, beef meat, and camel meat) were collected from retail meat shops in Zagazig City, Sharkia Governorate, Egypt. Samples were collected aseptically in sterile plastic bags between January to March 2022. Additionally, a total of 100 hand and stool swabs (50, each) were collected from workers at the retail shops where meat samples were collected. Hand swabs were collected by rolling a sterile swab moistened in buffered peptone water over the palmar surface of the workers’ hand. The swab was then placed in the collection tube containing buffered peptone water. Stool samples were collected in clean sterile cups, and a swab was taken from each sample and inserted in a collection tube containing buffered peptone water. All samples and swabs were transported in an icebox at 4 °C to the laboratory and processed within 12–24 h.

### ***E. coli*** isolation and identification

From each meat sample, 25 g were aseptically homogenized in 1:10 buffered peptone water and incubated for 24 h at 37 °C. Hand and stool swabs were also incubated in buffered peptone water. A loopful of incubated samples was then streaked onto Chromocult® Tryptone Bile X-glucuronide agar (Sigma Aldrich, Millipore, ISO 16,649) supplemented with 2 μg/mL of cefotaxime (CTX) and incubated for 24 h at 37 °C. Green blue colonies were picked and purified by streaking into Tryptone Soy Agar (TSA) (Merck, Darmstadt, Germany). The purified colonies were then identified by biochemical tests for indole, oxidase, catalase, Methlye-Red (MR), Voges-Proskauer (VP), citrate, urease, nitrate reduction and sugar fermentation.

To confirm the presence of *E. coli*, bacterial DNA from presumptive *E. coli* colonies was extracted using the QIAamp DNA Mini kit according to the manufacturer’s instructions (Qiagen GmbH, Hilden, Germany, Catalogue no. 51,304). Specific primers targeting the *phoA* gene (Table 1) were employed for amplification to confirm *E. coli* isolates [25].

**Table 1:** Primers, product size and annealing temperatures used for identification, virulence and antimicrobial resistance genes reported in the present study

Gene	Primer sequences (5’-3’)	Product size (bp)	Annealing (°C)	References
*hlyA*	fw: AACAAGGATAAGCACTGTTCTGGCT	1177	60˚C	[28]
	rev: ACCATATAAGCGGTCATTCCCGTCA			
*phoA*	fw: CGATTCTGGAAATGGCAAAAG	720 bp	60˚C	[25]
	rev: CGTGATCAGCGGTGACTATGAC			
*stx1*	fw: ACACTGGATGATCTCAGTGG	614	58˚C	[30]
	rev: CTGAATCCCCCTCCATTATG			
*stx2*	fw: CCATGACAACGGACAGCAGTT	779	58˚C	
	rev: CCTGTCAACTGAGCAGCACTTTG			
*ast*A	fw: CCATCAACACAGTATATCCGA	110	55˚C	[29]
	rev: GGTCGCGAGTGACGGCTTTGT			
*stx*2f	fw: AGA TTG GGC GTC ATT CAC TGG TTG	428	57˚C	[31]
	rev: TAC TTT AAT GGC CGC CCT GTC TCC			
*fim*H	fw: TGCAGAACGGATAAGCCGTGG	508	50˚C	[32]
	rev: GCAGTCACCTGCCCTCCGGTA			
*eae*A	fw: ATG CTT AGT GCT GGT TTA GG	248	51˚C	[33]
	rev: GCC TTC ATC ATT TCG CTT TC			
*bla*IMP	fw: CATGGTTTGGTGGTTCTTGT	488	53˚C	[39]
	rev: ATAATTTGGCGGACTTTGGC			
*bla*VIM	fw: AGTGGTGAGTATCCGACA	280	53˚C	
	rev: ATGAAAGTGCGTGGAGAC			
*bla*NDM	fw: GGCGGAATGGCTCATCACGA	287	55˚C	
	rev: CGCAACACAGCCTGACTTTC			
*bla*TEM	fw: ATCAGCAATAAACCAGC	516	54˚C	[40]
	rev: CCCCGAAGAACGTTTTC			
*bla*OXA-1	fw: ATATCTCTACTGTTGCATCTCC	619	54˚C	
	rev: AAACCCTTCAAACCATCC			
*bla*SHV	fw: AGGATTGACTGCCTTTTTG	392	54˚C	
	rev: ATTTGCTGATTTCGCTCG			
*bla*CMY	fw: GACAGCCTCTTTCTCCACA	1143	60˚C	[41]
	rev: TGGAACGAAGGCTACGTA			
*bla*CTX-M-1	fw: GTTACAATGTGTGAGAAGCAG	1041	60˚C	[42]
	rev: CCGTTTCCGCTATTACAAAC			
*tetA*(A)	fw: GGTTCACTCGAACGACGTCA	576	50˚C	[43]
	rev: CTGTCCGACAAGTTGCATGA			
*tetA*(B)	fw: CCTCAGCTTCTCAACGCGTG	633	50˚C	
	rev: GCACCTTGCTCATGACTCTT			
*sul*	fw: CGGCGTGGGCTACCTGAACG		60˚C	[44]
	rev: GCCGATCGCGTGAAGTTCCG			
*cml*A	fw: CCGCCACGGTGTTGTTGTTATC	698	50˚C	[45]
	rev: CACCTTGCCTGCCCATCATTAG			
*flo*R	fw: TTTGGWCCGCTMTCRGAC	494	50˚C	[46]
	rev: SGAGAARAAGACGAAGAAG			
*mcr-1*	fw: AGTCCGTTTGTTCTTGTGGC	320	58 °C	[47]
	rev: AGATCCTTGGTCTCGGCTTG			
*mcr-5*	fw: ATGCGGTTGTCTGCATTTATC	1644	58 °C	
	rev: TCATTGTGGTTGTCCTTTTCTG			
*chuA*	fw: GAC GAA CCA ACG GTC AGG AT	279	55˚C	[27]
	rev: TGC CGC CAG TAC CAA AGA CA			
*yjaA*	fw: TGA AGT GTC AGG AGA YGC TG	211		
	rev: ATG RAG AAT GCG TTC CTC AAC			
*tsp*E4C2	fw: GAG TAA TGT CGG GGC ATT CA	152		
	rev: CGC GYC AAC AAA GTA TTR CG			

### Characterization of ESBL-producing ***E. coli***

The Double Disc Synergy Test (DDST) was used to verify the confirmed *E. coli* strains for phenotypic ESBL expression [26].

### ESBL-producing ***E. coli*** phylotyping, virulotyping and genotyping

The confirmed strains were phylotyped using primers for the amplification of *chu*A, *yja*A, and, *tsp*E4C2 genes (Table 1), according to the phylotype classification scheme previously described [27].

Virulotyping was also performed using specific primers for amplification of the virulence associated genes (Table 1), including *hly* [28],, *ast*A [29], *stx*1 and *stx*2 [30], *stx*2f [31],, *fim*H [32], *eae*A [33].

Genotyping was performed on extracted DNA through fingerprinting PCR using REP-primers synthesized by Metabion (Germany) with the following sequences: Rep1R-I 5’- III ICG ICG ICA TCI GGC-3’ and Rep2-I 5’- ICG ICT TAT CIG GCC TAC-3’ [34]. The primers were included in a 25- μL reaction mixture containing 12.5 μL of EmeraldAmp Max PCR Master Mix (Takara, Japan), 1 μL of each primer ( 20 pmol), 4.5 μL of water, and 6 μL of DNA template. The PCR procedure was carried out using an Applied Biosystem 2720 thermal cycler.

To assess the discriminatory power of the REP-PCR fingerprinting data, the Simpson’s index of diversity (*D*) was utilized. The fingerprinting data was converted into a binary code, indicating the presence or absence of each band. A *D* value greater than 0.9 indicated good differentiation [35].

### Antimicrobial susceptibility testing

The Kirby-Bauer disc diffusion method was used to evaluate the isolates’ antibiotic susceptibility in accordance with the standards established by the National Committee for Clinical Laboratory Standards (NCCLS). Nineteen different antimicrobial agents were tested, and the zones of inhibition were measured and interpreted based on the guidelines provided by the Clinical and Laboratory Standards Institute (CLSI) [26]. In accordance with CLSI recommendations, the double fold dilution procedure (0.125-256 g/mL) was used to establish the minimum inhibitory concentration (MIC) for colistin [36]. The antimicrobial agents used included penicillin (PEN), ampicillin (AMP), amoxicillin (AMX), streptomycin (STR), erythromycin (ERY), nalidixic acid (NAL), amikacin (AMK), trimethoprim-sulfamethoxazole (SXT), kanamycin (KAN), neomycin (NEO), gentamicin (GEN), ciprofloxacin (CIP), tetracycline (TET), colistin (CST), imipenem (IPM), chloramphenicol (CHL), cefotaxime (CTX), ceftriaxone (CRO), ceftazidime (CAZ). *E. coli* ATCC 25,922 and *Staphylococcus aureus* ATCC 25,923 served as the microorganisms for quality control.

Multiple antibiotic resistance (MAR) index was calculated by dividing the number of antibiotics to which *E. coli* isolates showed resistance by the total number of drugs tested [37]. Multidrug resistance (MDR) was defined as the resistance of an isolate to at least one agent in three or more antibiotic classes [38].

### Antimicrobial resistance genes

Bacterial DNA from the *E. coli* confirmed isolates was also screened for ESBL encoding genes (Table 1); *bla*IMP, *bla*VIM, and *bla*NDM [39], *bla*TEM, *bla*OXA-1, and *bla*SHV [40], *bla*CMY [41], *bla*CTX-M-1 [42], tetracycline; *tetA*(A) and *tetA*(B) [43], sulfonamides; *sul* [44], chloramphenicol; *cml*A [45], and florquinolones; *flo*R [46]. The presence of colistin resistance genes *mcr-*1 to *mcr-*5 was also examined [47].

### Antimicrobial effect of silver nanoparticles on MDR ESBL-producing ***E. coli***

AgNPs-H_2_O_2_ (Top Superpower-vision) was obtained as a commercial product from El-Delta Center for Nanosilver Technology Company, Mansoura, Egypt. The stock solution of the product contained 45-nm silver nanoparticles (0.00004467 mL/liter), hydrogen peroxide (50% per liter) and natural herb mint (1 mL/liter) at a concentration of 5 mL/liter of water. The particles size was previously determined to be 30.17–67.92 nm with a zeta potential estimation of − 0.192 mV [48]. The AgNPs-H_2_O_2_ mixture was prepared by diluting the stock solution in sterile distilled water to achieve the desired commercial concentration. The minimum inhibitory concentrations (MIC_50_ and MIC_90_) of AgNPs-H_2_O_2_ were determined against MDR ESBL-*E. coli* by the broth microdilution method [36]. Briefly, microtiter plate wells were supplemented with various concentrations of AgNPs-H_2_O_2_ ranging from 100, 50, 25, 10, 5, 2.5 1.25, 0.625, 0.312, 0.156 and 0.078 μg/mL. MDR- ESBL-producing colonies were added in Muller Hinton broth and adjusted to the density of a 0.5 McFarland standard (1 × 10^8^ cfu/ml). Each well received a final inoculum of 5 × 10^5^ cfu/mL, and the plates were incubated for 24 h at 37 °C. A well with MHB alone and another well included MHB with AgNPs-H_2_O_2_ were used as reference control. The lowest agent concentration that entirely prevents an organism’s observable growth is known as the MIC endpoint. The MIC_50_ and MIC_90_ were calculated using an orderly array method [49], where the middle value was selected as MIC_50_. The MIC_90_ was determined in the same way by selecting the appropriate value from the orderly array.

### Data analysis

For statistical analysis and data visualization, R software was used (R Core Team, 2022; version 4.2.0). The *E. coli* isolation rate was calculated by dividing the number of *E. coli* positive samples by the total number of samples tested for each source. Pearson’s chi-square test was employed to assess any variations in the *E. coli* isolation rates among different sample types. The heatmap was created using the “Complex heatmap” package [50], and the dendrogram was created using the “hclust” function of the stats package. Furthermore, one-way analysis of variance was conducted to compare the MAR index of isolates from different sources. *P*-values < 0.05 were considered statistically significant.

## Results

### ***E. coli*** isolation and identification

Out of the 350 samples tested, 112 (32%) were *E. coli* positive with 68.75% (77/112) were isolated from retail meat samples and the remaining 31.25% (35/112) were recovered from retail market workers. *E. coli* was identified in various types of retail meat and market workers samples. The *E. coli* isolation rates exhibited significant variation (*P* = 0.0003) among the different sample sources. The highest isolation rate (54%) was observed in beef meat samples followed by workers stool samples (48%) (Table 2).

**Table 2:** Proportion of *E. coli* and ESBL-producing *E. coli* isolated from retail meat samples and retail shop workers

Source	Sampling site	Number examined	No. of *E. coli* positive (%)	No. of ESBL positive *E. coli* (%)	Phylogenetic group	
					A	B1
Chickens	Breast meat	50	17 (34%)	10 (58.8%)	0	10 (100%)
Ducks	Breast meat	50	11 (22%)	9 (81.8%)	0	9 (100%)
Turkey	Breast meat	50	9 (18%)	5 (55.6%)	0	5 (100%)
Beef	Cube meat	50	27 (54%)	13 (48.1%)	0	13 (100%)
Camel	Cube meat	50	13 (26%)	8 (61.5%)	0	8 (100%)
Workers	Hand swabs	50	11 (22%)	9 (81.8%)	0	9 (100%)
	Stool swabs	50	24 (48%)	11 (45.8%)	4 (36.4%)	7 (63.6%)
**Total**		**350**	**112 (32%)**	**65 (58%)**	**4 (6.2%)**	**61 (93.8%)**

### ESBL-producing ***E. coli***

Overall, 58% (65/112) of *E. coli* isolates were ESBL producers, with 45 (69.2%) and 20 (30.8%) isolates recovered from retail meat and market workers samples, respectively (Table 2). The highest proportion of ESBL-producing *E. coli* was observed in duck meat and worker hand swabs (81.8%), followed by camel meat samples (61.5%).

### Phylotyping, virulotyping and genotyping

**Fig. 1:**
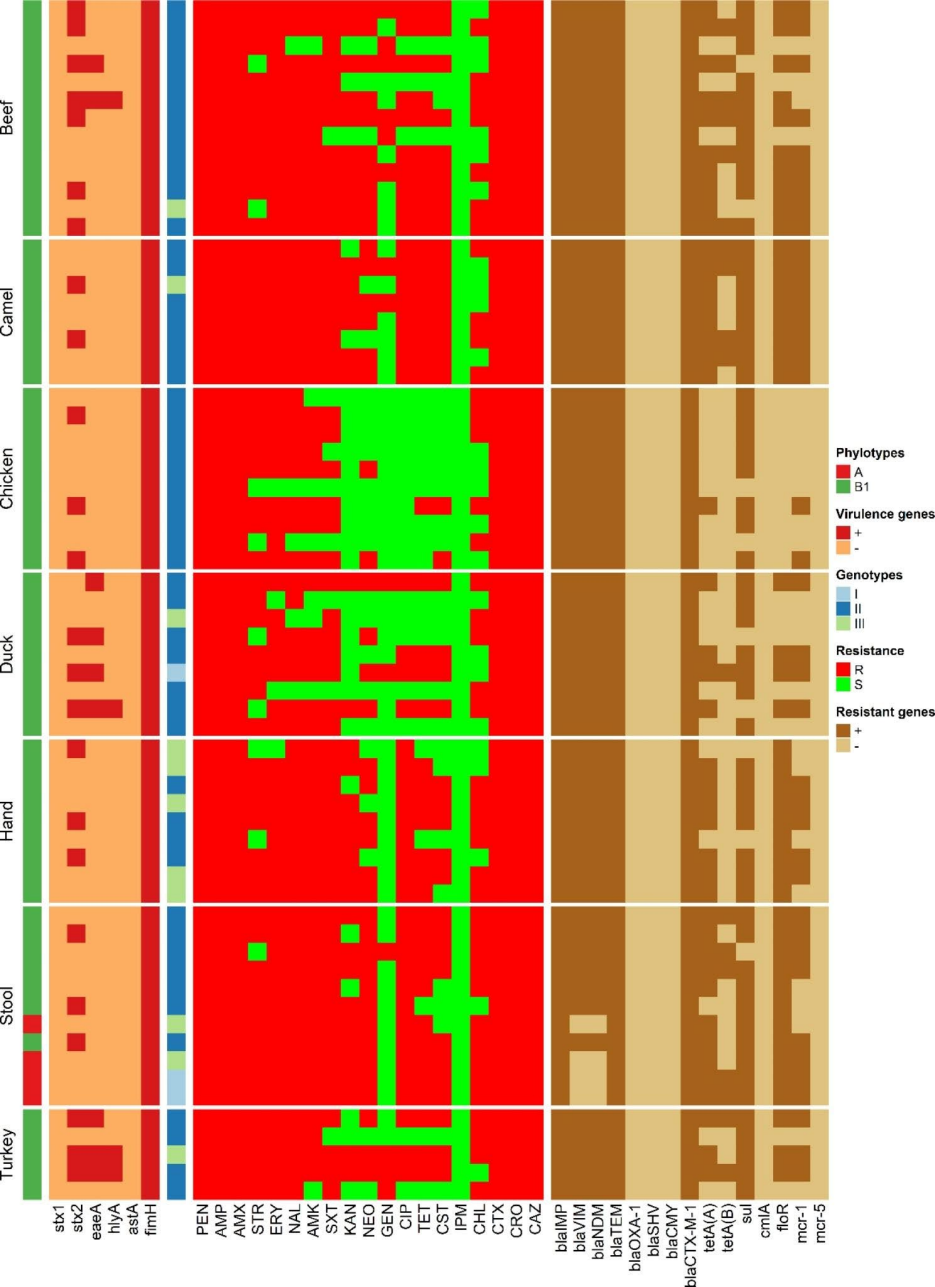
Heatmap representation of ESBL-producing *E. coli* phylotypes, virulotypes, genotypes, antimicrobial resistance patterns and genes

**Fig. 2:**
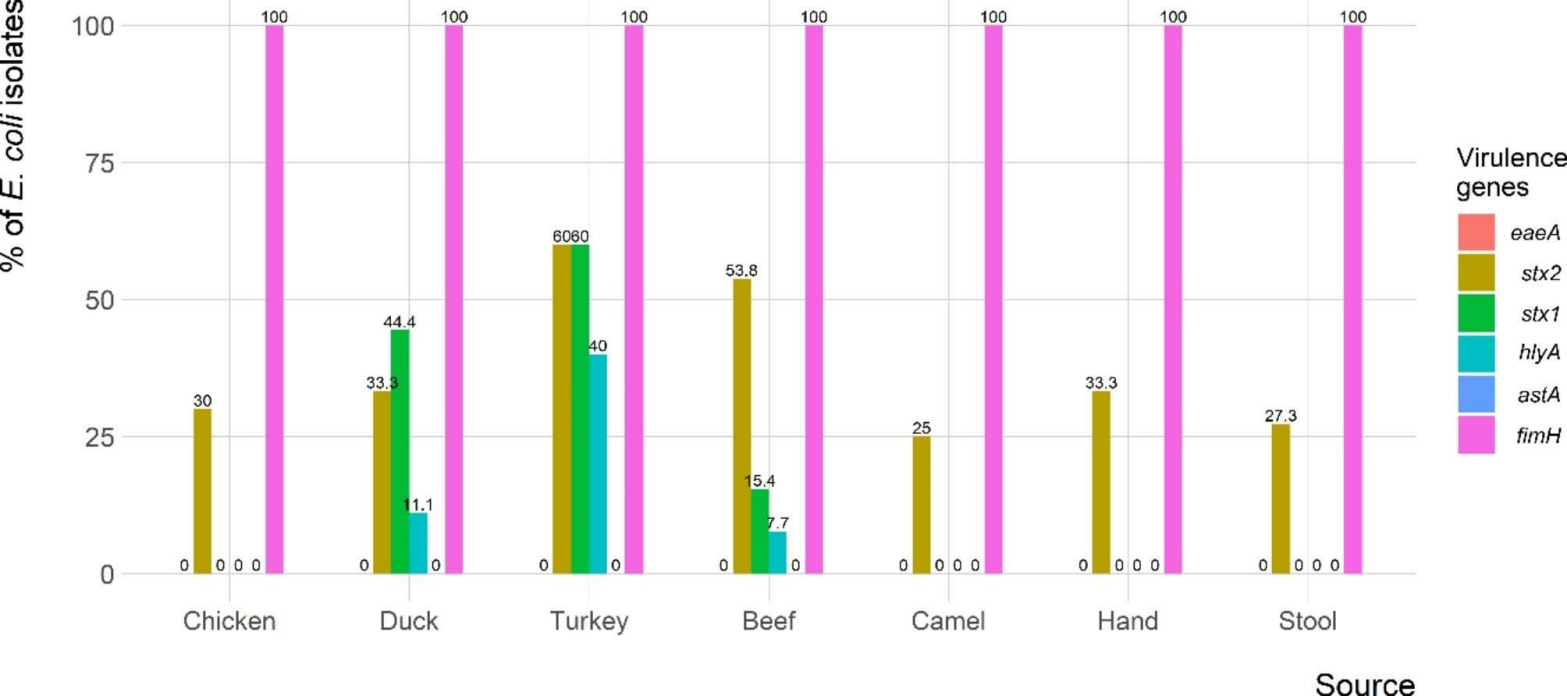
Frequency of virulence genes of ESBL-producing *E. coli* isolated from retail meat samples and retail shop workers

The phylogenetic grouping of the 65 ESBL-producing *E. coli* showed that 4 (6.2%) isolates belonged to group A and 61 (93.8%) to group B1 (Table 2). All group A isolates were recovered from workers stool samples (Fig. 1). None of the isolates belonged to group B2 or D. All the ESBL-producing *E. coli* isolates (100%) harbored at least one virulence gene, but only 25 (38.5%) of the isolates harbored two or more virulence genes (Fig. 2). The *stx*2 gene was amplified in 24 (36.9%) of the isolates, followed by *eae*A and *hly*A genes in 9 (13.8%) and 4 (6.2%) isolates, respectively. The *stx*2f gene was identified in only four isolates that tested positive for *stx*2 gene (one each from chicken, turkey, duck, and beef meat samples). None of the isolates carried the *ast*A or *stx*1genes, however, all of them tested positive for the *fim*H gene (Fig. 2).

**Fig. 3:**
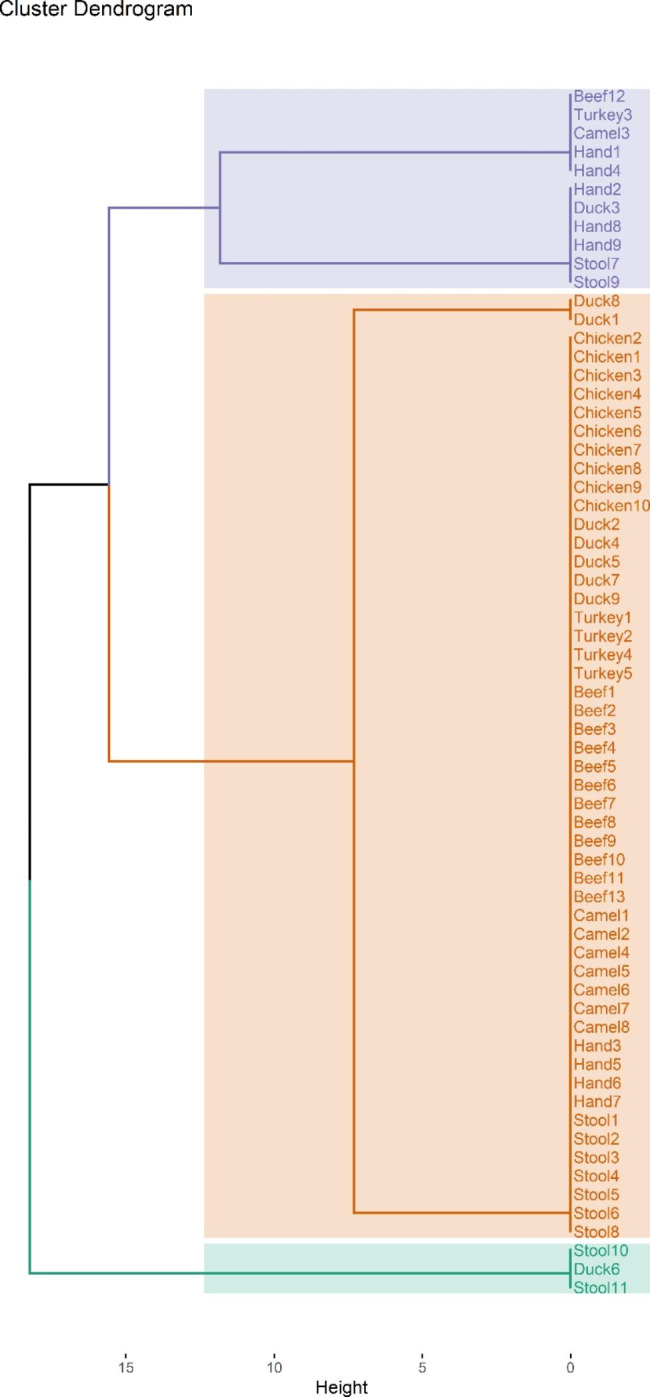
REP-PCR based dendrogram for ESBL-producing *E. coli* isolated from retail meat samples and retail shop workers

A single amplification profile was used to analyze the REP-PCR patterns of the 65 ESBL-producing *E. coli* isolates. The profiles were distinguished based on the number and position of the amplified fragments, which ranged in size from 290 to 1600 bp. Visual examination of the banding patterns revealed that five profiles (E1 to E5) were generated (Fig. 3). Simpson’s index of diversity was used to evaluate the discriminatory power of the REP-PCR, and the results showed that it had relatively low discriminatory power with a *D* value of 0.42. There were three main clusters revealed by the dendrogram analysis of the 65 analyzed isolates. (Figures 1 and 3). Notably, isolates from workers and meat within the same cluster exhibited 100% similarity.

### Antimicrobial susceptibility testing

**Fig. 4:**
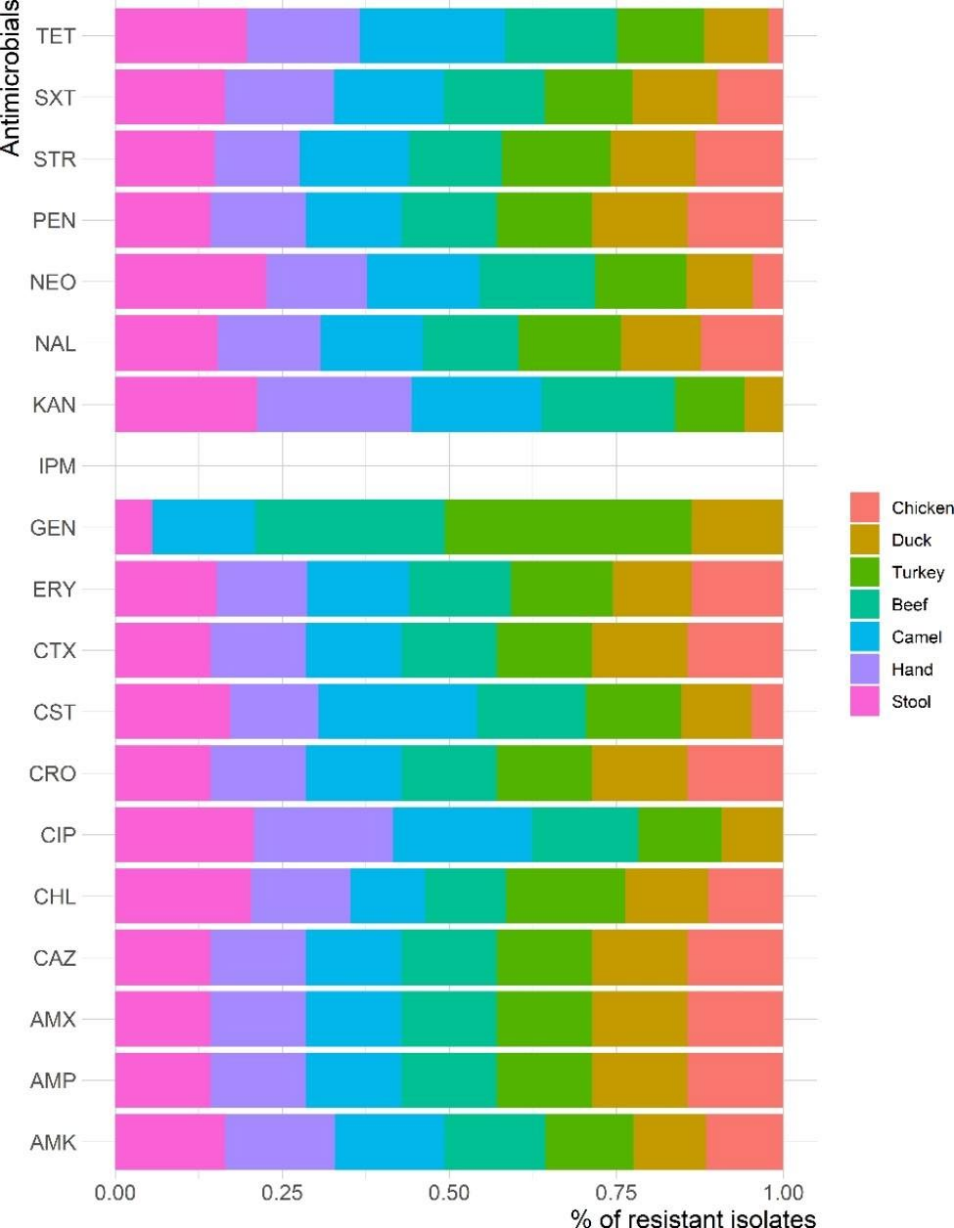
Frequency of antimicrobial resistance of ESBL-producing *E. coli* isolated from retail meat samples and retail shop workers

**Fig. 5:**
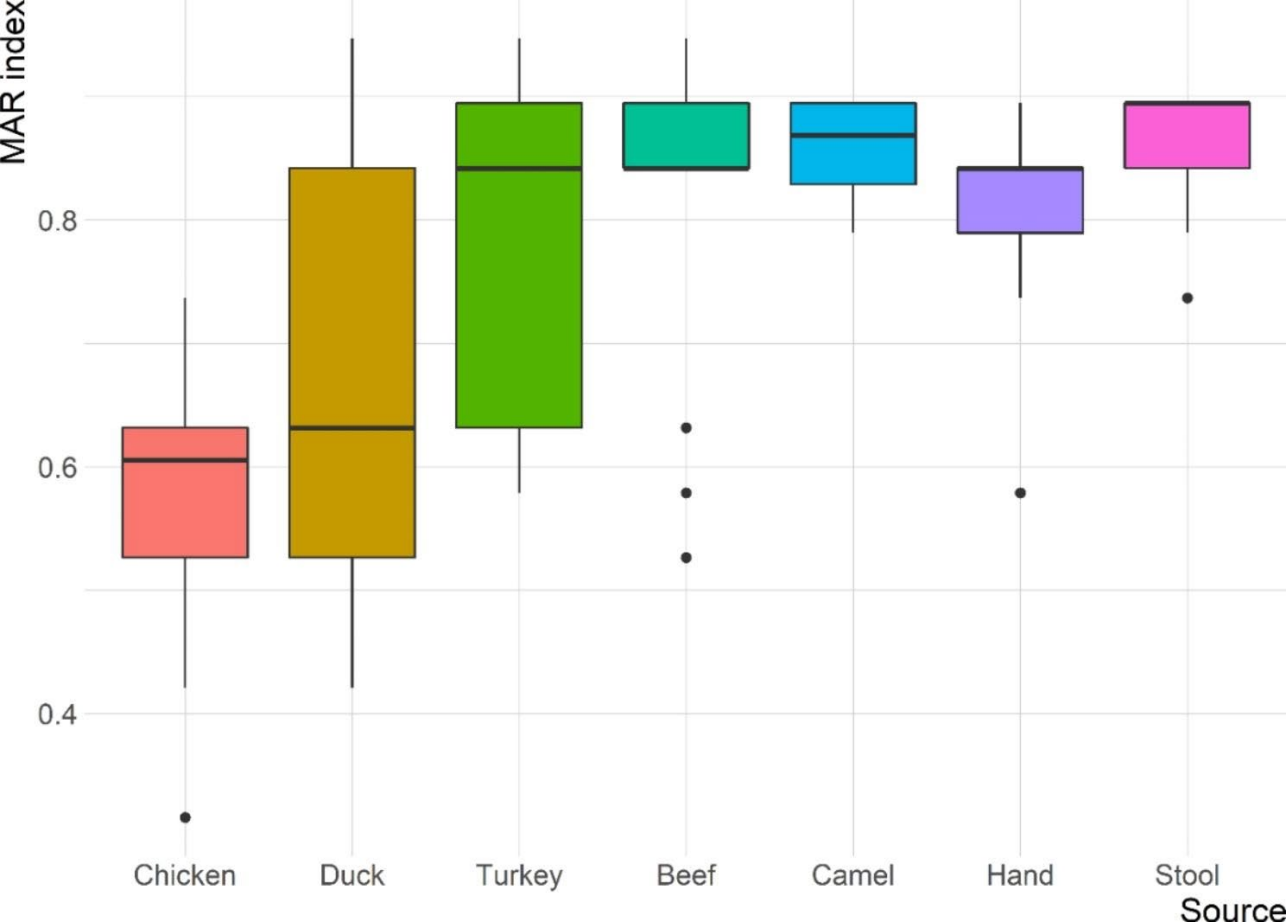
Multiple antibiotic resistance (MAR) index box plot of ESBL-producing *E. coli* isolated from retail meat samples and retail shop workers

Table 3 presents the antimicrobial susceptibility profiles of the 65 ESBL-producing *E. coli* isolates. The isolates showed high frequencies of resistance to PEN, AMP, AMX, CTX, CAZ and CRO (100%, each), while the lowest resistance rate (21.5%) was observed for GEN. All isolates were susceptible to IMP. Figure 4 displays the frequency of antimicrobial resistance by source of isolation. However, no significant difference (*P* > 0.05) was found between resistance rates of ESBL-producing *E. coli* isolated from retail meat and market workers. All isolates were classified as MDR with MAR index ranging from 0.32 to 0.95 and an average of 0.70 (Fig. 5). The average MAR index of isolates recovered from various sources differed significantly, with workers’ stool having the highest MAR index (0.86) followed by camel meat (0.85).

**Table 3:** Frequency of antimicrobial resistance profiles of ESBL-producing *E. coli* isolated from retail meat samples and retail shop workers

Antimicrobial resistance profiles^1^	Frequency of *E. coli* isolates from each source							Total
	Chicken	Duck	Turkey	Beef	Camel	Hand	Stool	
PEN, AMP, AMX, STR, ERY, NAL, AMK, SXT, KAN, NEO, CIP, TET, CST, CHL, CTX, CRO, CAZ				2	2	2	6	12
PEN, AMP, AMX, STR, ERY, NAL, AMK, SXT, KAN, NEO, GEN, CIP, TET, CST, CHL, CTX, CRO, CAZ		1	1	2				4
PEN, AMP, AMX, STR, ERY, NAL, AMK, SXT, KAN, NEO, GEN, CIP, TET, CST, CTX, CRO, CAZ			1	1	2			4
PEN, AMP, AMX, STR, ERY, NAL, AMK, SXT, NEO, CIP, TET, CST, CHL, CTX, CRO, CAZ			1		1	1	1	4
PEN, AMP, AMX, STR, ERY, NAL, AMK, SXT, KAN, NEO, CIP, TET, CST, CTX, CRO, CAZ				2	1			3
PEN, AMP, AMX, STR, ERY, NAL, AMK, SXT, KAN, NEO, CIP, TET, CHL, CTX, CRO, CAZ				1		1	1	3
PEN, AMP, AMX, STR, ERY, NAL, AMK, SXT, CHL, CTX, CRO, CAZ	2			1				3
PEN, AMP, AMX, ERY, NAL, AMK, SXT, KAN, NEO, CIP, TET, CST, CHL, CTX, CRO, CAZ		1		1				2
PEN, AMP, AMX, STR, ERY, NAL, AMK, SXT, KAN, CIP, TET, CST, CTX, CRO, CAZ					1	1		2
PEN, AMP, AMX, STR, ERY, NAL, AMK, SXT, CTX, CRO, CAZ	1	1						2
PEN, AMP, AMX, ERY, NAL, AMK, SXT, KAN, NEO, GEN, CIP, TET, CST, CHL, CTX, CRO, CAZ							1	1
PEN, AMP, AMX, ERY, NAL, AMK, SXT, KAN, NEO, GEN, CIP, TET, CST, CTX, CRO, CAZ				1				1
PEN, AMP, AMX, STR, ERY, NAL, AMK, SXT, KAN, CIP, TET, CST, CHL, CTX, CRO, CAZ						1		1
PEN, AMP, AMX, STR, ERY, NAL, AMK, SXT, NEO, GEN, CIP, TET, CST, CTX, CRO, CAZ		1						1
PEN, AMP, AMX, STR, ERY, NAL, AMK, SXT, CIP, TET, CST, CHL, CTX, CRO, CAZ					1			1
PEN, AMP, AMX, STR, ERY, NAL, AMK, SXT, KAN, NEO, CIP, TET, CTX, CRO, CAZ						1		1
PEN, AMP, AMX, STR, ERY, NAL, AMK, SXT, NEO, CIP, TET, CHL, CTX, CRO, CAZ							1	1
PEN, AMP, AMX, ERY, NAL, AMK, SXT, KAN, NEO, CIP, CHL, CTX, CRO, CAZ						1		1
PEN, AMP, AMX, STR, ERY, NAL, AMK, SXT, CIP, TET, CST, CTX, CRO, CAZ		1						1
PEN, AMP, AMX, STR, ERY, NAL, AMK, SXT, KAN, NEO, CIP, CTX, CRO, CAZ							1	1
PEN, AMP, AMX, STR, ERY, NAL, AMK, SXT, TET, CST, CHL, CTX, CRO, CAZ	1							1
PEN, AMP, AMX, STR, ERY, NAL, AMK, SXT, NEO, CST, CTX, CRO, CAZ	1							1
PEN, AMP, AMX, ERY, NAL, AMK, SXT, NEO, CHL, CTX, CRO, CAZ		1						1
PEN, AMP, AMX, STR, ERY, NAL, AMK, SXT, NEO, CTX, CRO, CAZ	1							1
PEN, AMP, AMX, STR, ERY, NAL, SXT, GEN, CHL, CTX, CRO, CAZ			1					1
PEN, AMP, AMX, NAL, AMK, SXT, KAN, CIP, CTX, CRO, CAZ						1		1
PEN, AMP, AMX, STR, ERY, NAL, AMK, CHL, CTX, CRO, CAZ			1					1
PEN, AMP, AMX, STR, ERY, NAL, AMK, GEN, CTX, CRO, CAZ				1				1
PEN, AMP, AMX, STR, ERY, NAL, AMK, CTX, CRO, CAZ	1							1
PEN, AMP, AMX, STR, ERY, NAL, CHL, CTX, CRO, CAZ	1							1
PEN, AMP, AMX, STR, ERY, SXT, CHL, CTX, CRO, CAZ		1						1
PEN, AMP, AMX, STR, ERY, SXT, GEN, CTX, CRO, CAZ				1				1
PEN, AMP, AMX, ERY, CHL, CTX, CRO, CAZ	1							1
PEN, AMP, AMX, STR, CHL, CTX, CRO, CAZ		1						1
PEN, AMP, AMX, STR, NAL, CTX, CRO, CAZ		1						1
PEN, AMP, AMX, CTX, CRO, CAZ	1							1

^1^ PEN, penicillin; AMP, ampicillin; AMX, amoxicillin; STR, streptomycin; AMK, amikacin; KAN, kanamycin; NEO, neomycin; GEN, gentamicin; SXT, trimethoprim-sulfamethoxazole; ERY, erythromycin; NAL, nalidixic acid; CIP, ciprofloxacin; TET, tetracycline; CST, colistin; IPM, imipenem; CHL, chloramphenicol; CTX, cefotaxime; CRO, ceftriaxone; CAZ, ceftazidime

### Antimicrobial resistance genes

**Fig. 6:**
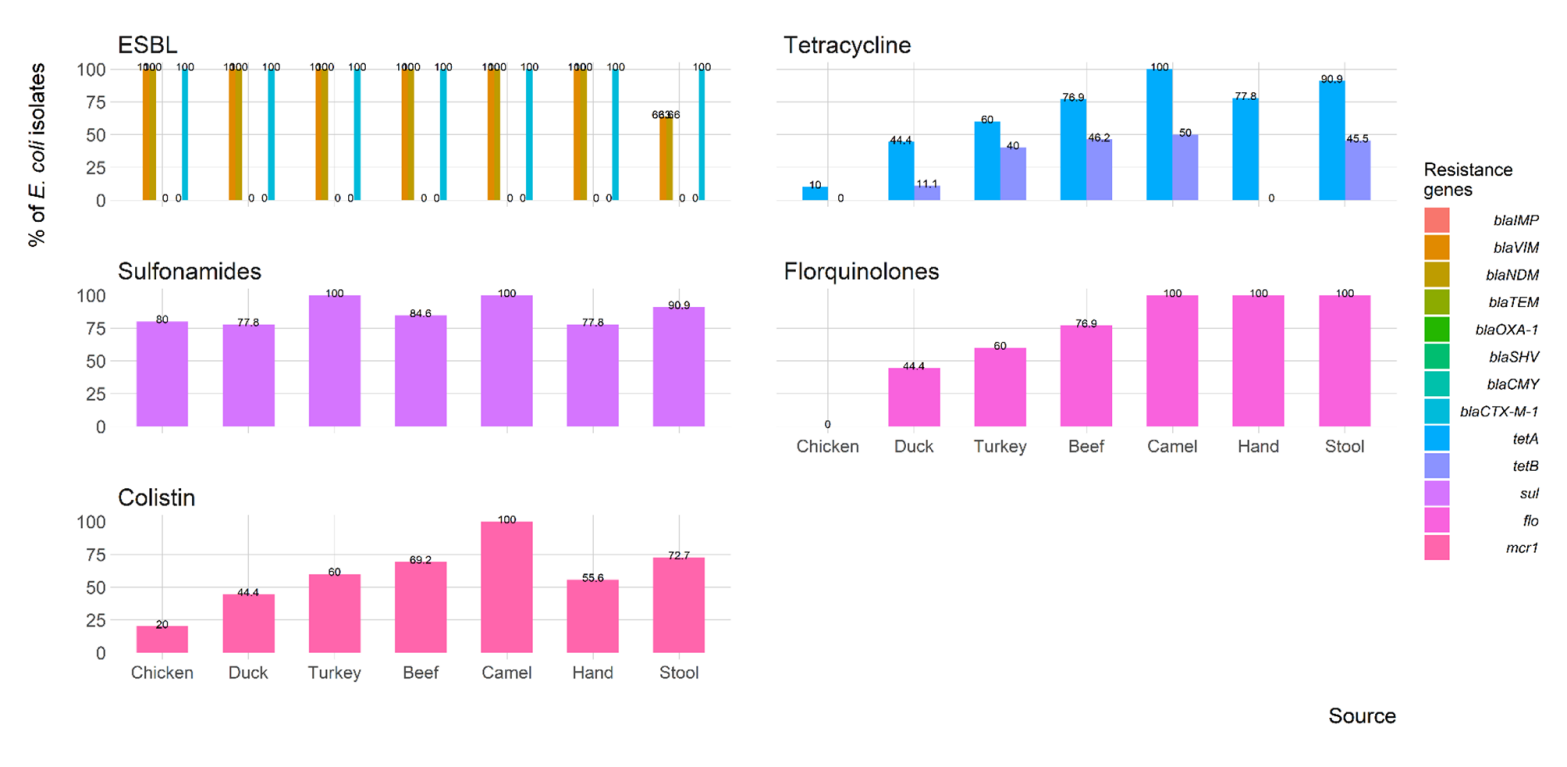
Frequency of antimicrobial resistance genes of ESBL-producing *E. coli* isolated from retail meat samples and retail shop workers

The frequency of antimicrobial resistance genes detected in ESBL-producing *E. coli* isolates obtained from retail meat and market workers is shown in Fig. 6. The ESBL encoding genes (*bla*IMP, *bla*TEM, and *bla*CTX-M-1) were found in 100% of the isolates, while *bla*VIM, and *bla*NDM were detected in 93.8% of the isolates. However, none of the isolates were positive for *bla*OXA-1, *bla*SHV, or *bla*CMY. Tetracycline resistance genes (*tet*A and *tet*B) were detected in 66.2% and 27.7% of the tested isolates, respectively The resistance genes for sulfonamides (*sul*), and florquinolones (*flo*R) were identified in 86.2% and 69.2% of the isolates, respectively. Only *mcr*-1 was detected among the tested colistin resistance genes (*mcr*-1 and *mcr*-5), and it was found in 60% of the isolates.

### Antimicrobial effect of silver nanoparticles

The antimicrobial activity of AgNPs-H_2_O_2_ against ESBL-producing *E. coli* was evaluated using the broth microdilution method. The MIC values of different concentrations of AgNPs-H_2_O_2_ against ESBL-producing *E. coli* isolated from retail meat and market workers are illustrated in Table 4. AgNPs-H_2_O_2_ concentrations of 0.625, 1.25, 2.5 and 5 μg/mL showed complete bacterial growth inhibition (no turbidity). The MIC_50_ and MIC_90_ were 0.625 and 2.5 μg/mL, respectively.

**Table 4:** The distribution of minimum inhibition concentration (MIC) values of AgNPs concentrations against ESBL-producing *E. coli* isolated from retail meat samples and retail shop workers

Source	No. of sensitive *E. coli* isolate at different AgNPs concentrations (μg/mL)						
	5	2.5	1.25	0.625	0.312	0.156	0.078
Chickens	2	2	0	6	0	0	0
Ducks	0	3	1	5	0	0	0
Turkey	0	2	0	3	0	0	0
Beef	3	1	0	9	0	0	0
Camel	0	3	1	4	0	0	0
Worker’s hand	0	1	2	6	0	0	0
Worker’s stool	0	0	2	9	0	0	0
**Total**	**5**	**12**	**6**	**42**	**0**	**0**	**0**

## Discussion

Unhygienic food handling and processing procedures facilitate the dissemination of MDR bacteria, including ESBL-*E. coli* to human consumers. Thus, enforcing strict monitoring measures and promoting sanitary procedures for meat distribution are necessary to prevent the proliferation of antibiotic-resistant bacteria [4].

In this study, ESBL-*E. coli* prevalence in duck meat was higher compared to the other sources (81.8%), followed by camel meat (61.5%). Similar results were reported in other studies in China [51] and Thailand [52], where MDR *E. coli* isolates were more common in ducks than in chicken. The higher isolation rate of ESBL-*E. coli* from ducks could be attributed to their nature as waterfowl, which excretes feces in water, thus enhancing the spread of the pathogens within the duck population [52].

A high ESBL-producing *E. coli* isolation rate of 86.7 from chicken meat in Turkey was reported by Kürekci, et al. [53]. The authors attributed the high isolation rate from chicken meat due to the extensive use of fluoroquinolones, cephalosporines and aminoglycosides in poultry industry which resulted in selection pressure for the high carriage rate of ESBL-producing *E. coli* in chickens [54].

The prevalence of ESBL-producing *E. coli* in beef was lower than that in chicken, which is consistent with the findings of Randall, et al. [55] who found rates of 20% in beef samples compared to 63% in chicken samples. Rao, et al. [56], also reported that only 1.9% of beef samples were positive for ESBL-producing *E. coli*, whereas 65.4% of chicken samples tested positive. Meanwhile, the prevalence of ESBL-producing *E. coli* in beef samples in the current study (48.1%) was much higher than the average (7.1%) of ESBL/AmpC-producing *E. coli* found in beef samples purchased at retail in the EU [57].

Meat is highly susceptible to microbial contamination due to multiple contacts from the slaughterhouse until consumption as food [4]. The prevalence of ESBL-*E. coli* was found to be high in camel (61.5%) and beef meat (48.1%) samples, this is in line with another study [4]. Similarly, El-Ghareeb, et al. [58] reported the isolation of ESBL-producing *E. coli* from 11.3% camel minced meat samples in Saudi Arabia. The high water and protein content of red meat provide a favorable environment for bacterial growth and may contribute to the high contamination rates [59].

In the present study, ESBL-*E. coli* was found to be prevalent in workers hand swabs (81.8%) and stool samples (45.8%). Similar findings were reported in studies conducted in Thailand [52, 60]. In Ethiopia, *E. coli* was isolated from 20% of hand swabs, with ESBL-*E. coli* accounting for 25% of the isolates [4]. Variations in the hygienic procedures of meat handlers and the sanitary standards of meat retail shops may be the cause of the observed differences in *E. coli* prevalence. Inadequate hygienic procedures of meat handlers and insufficient sanitation standards in meat retailer stores may lead to cross-contamination of meat with *E. coli* [4].

*E. coli* can be classified into four main phylogenetic groups named A1, B1, B2, and D, which can be identified by PCR of four genes [6]. Generally, commensal strains belong to groups A1 and B1, while most virulent strains belong to groups B2 and D [6, 61]. In the present study, all the isolates were of the commensal groups A (6.2%) and B1 (93.8). Previous studies have also reported that the majority of the *E. coli* phylotypes from different sources were of the commensal groups A1 and B1 [61–63]. In Egypt, Abdallah, et al. [64] reported that 80% of ESBL-producing *E. coli* isolated from chicken meat in the same study area were also of the commensal groups. The high proportion of commensal strains highlight their critical silent role in the spread and dissemination of ESBL-resistance genes [61, 64].

Virulotyping of the isolates revealed that all had at least one virulence gene, with *stx*2 (36.9%) being the most frequently identified gene, and four isolates being positive for *stx*2f gene. The existence of genes related to virulence such as *stx*1, *stx*2, and *eae* have been found to be crucial factors for the pathogenicity of *E. coli* strains [65]. *Stx*2-encoding strains of Shiga toxin producing *E. coli* (STEC) have been linked to more severe infections than those only possessing *stx*1 [66]. In our study, *stx*2 was the most common virulence gene (36.9%), consistent with reports from Germany, Argentina, and China [67–69]. In line with our findings, a study conducted in Egypt reported that 36% of MDR *E. coli* isolates obtained from chicken meat carried the *stx*2 gene [70].

In China, 28.57% and 51.02% of *E. coli* isolates had *stx*1 and *stx*2 genes, respectively [69]. Although Shiga toxin is necessary for STEC pathogenicity, it is not sufficient. Therefore, we examined four additional virulence-associated genes: *hly, ast, eae*, and *fim*H, that are linked to bacterial virulence. The combination of the *eae* and *stx* genes has been associated with increased virulence [71]. None of the isolates in our investigation carried the *ast*A or *stx*1genes, while Nong, et al. [69] found *ast*A in 20.41% and Ali, et al. [70] detected *stx*1 gene in 4% of the *E.coli* strains from chicken meat. All tested isolates in our study were positive for *fim*H gene, which is responsible for adhesion (the first step in the colonization process). Similar results were reported in poultry meat from Brazil [53, 72]. This is expected because this gene has been reported from both clinical and commensal *E. coli* isolates from different sources [53, 57]. The *eae*A and *hly*A genes were found in 13.8% and 6.2% of our isolates, respectively. A lower proportion of *eae*A and *hly*A encoding genes (5 and 4%, respectively) among *E. coli* isolates was reported previously in Egypt by Ali, et al. [70]. However, a higher percentage of *eae* -encoding genes (34.69%) among the *E. coli* isolates was observed in China [69]. In contrast, none of ESBL-producing *E. coli* recovered from retail meat in Mexico were positive of *stx*1, *stx*2, *hly*A, or *eae*, virulence genes [61].

Recent studies highlight the significance of comparing isolates from various sources to evaluate the relevance of the foodborne pathway in human infection [73]. This study utilized REP-PCR to investigate the genetic relatedness between the isolates from different sources. The isolates were grouped into five profiles in three clusters. The 100% similarity between human and meat isolates within the same cluster indicates genetic relatedness and the possibility of transmission of strains from meat to humans. This is supported by the isolation of the same ESBL-associated genes from human and meat isolates. A similar study conducted in the Netherlands also reported a partially close genetic relationship between strains obtained from human carriers and chicken meat samples [74]. A Japanese study also reported 80% similarity index between *E. coli* isolates harboring *β*-lactamase obtained from domestic and imported chicken meat samples [75]. Similarly, a study in Sweden revealed that less than 0.1% of the population carried ESBL-producing isolates associated with poultry, but 5% of individuals were colonized with ESBL-encoding plasmids that were identical to those found in chicken meat and poultry isolates [76]. Additionally, a study conducted in rural Ghana demonstrated genetic links between ESBL-producing *E. coli*, suggesting possible transmission between poultry and human populations [77].

However, Belmar Campos, et al. [21] reported that ESBL genes produced by *E. coli* from chicken meat are different from those found in human stool samples and their data do not support the notion that ESBL strains from chicken meat significantly contribute to human colonization. The authors attributed this discrepancy to the fact that the chicken meat samples were collected more than six months after the human feces samples were obtained. Similarly, other studies have reported no genetic relatedness between ESBL-producing isolates from food animals and humans in close contact [73, 76]. In a study conducted in the Netherlands, no evidence was found to support the hypothesis of clonal transmission of ESBL-producing *E. coli* isolates between humans and poultry [78].

All isolates tested in our study were found to be MDR, with an average MAR index ranging from 0.32 to 0.95. The isolates showed 100% resistance to penicillins, beta-lactams and cephems. The MDR isolates are considered reservoirs for both resistance and virulence genes and can be transferred to other strains of the same species and other species, thereby increasing the source of antibiotic resistance [61]. Similar results were reported by other studies [52, 55]. Abayneh, et al. [4] reported that the majority of *E. coli* isolates (74.3%) exhibited resistance to three or more classes of antibiotics, including TET, ERY and cotrimoxazole. Additionally, 85.7% of ESBL-producers were resistant to CTX and CRO, while 71.4% were resistant to CAZ. Another study reported high resistance of ESBL-*E. coli* to AMP (69.4%), SXT (66.7%), TET (88.9%) and Sulfonamide (75%) [79]. In China, ESBL-producing *E. coli* were found to be resistant to different antibiotics such as AMP (98.9%) and TET (97.6%) [80]. Another study in Egypt TET (80.9%), STR (61.9%) and SXT (61.9%) [81]. All our isolates were susceptible to imipenem, this is consistent with another study conducted in Turkey on chicken meat samples [53]. The difference between the resistance rates of ESBL-producing *E. coli* to antimicrobials could be attributed to antibiotic administration practices in the veterinary field as growth promoters or for therapy, also due to geographic distribution [3].

Colistin resistance has been observed in *E. coli* isolates from food-producing animals, especially poultry [82]. In this study, 60% of the isolates were resistant to colistin. The resistance to colistin is mediated by chromosomal mutations in *pmr*A/B, *pho*P/Q, and *mgr*B genes. However, an acquired colistin-resistance gene *mcr*-1 has also been identified in *E. coli* [83, 84]. To date, nine variants of *mcr* have been identified in humans and different animals [85]. The existence of *mcr* genes in mobile genetic elements raises concerns about their potential horizontal transfer in the food chain, posing a risk to public health [14]. Since 2006, the coexistence of *mcr* genes with other resistance determinants, such as ESBL and/or carbapenemase genes, has been reported in *Enterobacterales*. Notably, an increase in the prevalence of *mcr*-1 genes has been observed among ESBL-producing *E. coli* strains in animals, while their occurrence remains low in non-ESBL-producing *E. coli* strains. This suggests that the use of extended-spectrum cephalosporins may have contributed to the dissemination of *mcr*-1 [86, 87]. A study conducted in Turkey revealed a close genetic relationship between *mcr*-1 genes found in chicken meat and isolates of human origin, indicating the emergence and spread of *mcr*-mediated colistin resistance in *E. coli* across various sources with zoonotic potential in the food chain [88].

In this study, none *β*-lactamase resistance genes including *tet*A, *tet*B, *sul* and *flor* were identified with high isolation rates ranging from 27.7% to 86.2%. In the same line, a previous study has reported high prevalence of *tet*A (72.58%), and *sul*1 (44.67%) [89]. The investigation of resistance determinants in our study indicated that ESBL-encoding genes were highly prevalent in the isolates. TEM, SHV, OXA, CMY, and CTX-M beta-lactamases are the most prevalent beta-lactamases in Gram-negative bacteria. In accordance, a study in Egypt reported that 57.55%, 46.23%, and 23.58% of the isolates had TEM, CTX-M, and SHV genes, respectively [64], In Bangladesh, Rahman, et al. [89] reported only *bla*SHV gene from ESBL-*E. coli* isolates, while in the Netherlands, *bla*CTX-M-1(58.1%) is the most prevalent gene found in chicken meat, followed by *bla*TEM-52 (14%) and *bla*SHV-12 (14%) [90]. In Singapore, Guo, et al. [91] found that out of 225 ESBL-producing *E. coli* isolates, 76.4% carried *bla*CTX-M genes, 45.3% carried *bla*TEM genes and 23.1% carried *bla*SHV genes. Additionally, Lim, et al. [92] emphasized the prevalence of CTX-M genes as ubiquitous ESBL genes in ESBL-producing *E. coli*.

Although our isolates were sensitive to imipenem by phenotypic test, carbapenem resistance genes were detected in the isolates by PCR. This indicates that not all carbapenemase-producing isolates exhibit phenotypic resistance to carbapenems due to either lack of expression or the level of gene expression is less than the required to exhibit phenotypic resistance [93, 94].

Treatment of infections caused by ESBL-producing *E. coli* requires high doses of antibiotics, which can lead to antibiotic resistance and adverse effects on patients [95]. Nanoparticles, such as silver nanoparticles, are considered alternatives to antibiotics for treating various infections. Silver nanoparticles have large surface area to volume ratio, allowing for increased contact with bacteria and resulting in direct interaction with the bacterial cell wall to produce antibacterial activity [96]. Our results showed that the MIC_50_ of AgNPs-H_2_O_2_ was 0.625 μg/mL against ESBL-producing *E. coli*. Another study in Egypt reported that the average MIC value of AgNPs against ESBL-producing *E. coli* was 27 μg/ml [95]. In India, an average MIC values of 11.25-45 μg/mL was reported [97],[103] while in Mexico, an MIC of 10 μg/mL was demonstrated for AgNPs [98]. The discrepancies in MIC values could be attributed to differences in the particle size of AgNPs [95]. Shafreen, et al. [99] argued that silver nanoparticles suspensions prepared by biological methods and with concentrations higher than 100 μg/mL may lose their antibacterial effect on microorganisms.

## Conclusions

Results of present study showed high prevalence of virulent MDR ESBL-producing *E. coli* in retail meat products and workers in retail meat shops in Egypt. Therefore, regular monitoring of retail meat and application of hygienic food safety practices by food handlers are required for protecting consumers. Silver nanoparticles are considered a promising alternative for treating MDR ESBL-producing *E. coli* infections and reducing the risk of antimicrobial resistance.

## Data Availability

The entirety of the data generated or analyzed during this study has been included in the published article.
